# Emotion dysregulation in ADHD and other neurodevelopmental conditions: a co-twin control study

**DOI:** 10.1186/s13034-022-00528-0

**Published:** 2022-11-28

**Authors:** Rebecka Astenvald, Matilda A. Frick, Janina Neufeld, Sven Bölte, Johan Isaksson

**Affiliations:** 1 Department of Medical Sciences, Child and Adolescent Psychiatry Unit, Uppsala University, Uppsala, Sweden; 2 Department of Psychology, Division of Emotion Psychology, Uppsala University, Uppsala, Sweden; 3 Center of Neurodevelopmental Disorders (KIND), Centre for Psychiatry Research, Department of Women’s and Children’s Health, Karolinska Institutet & Stockholm Health Care Services, Stockholm, Sweden; 4 Swedish Collegium for Advanced Study (SCAS), Uppsala, Sweden; 5 Curtin Autism Research Group, Curtin School of Allied Health, Curtin University, Perth, WA Australia; 6 Child and Adolescent Psychiatry, Stockholm Health Care Services, Stockholm, Sweden

**Keywords:** ADHD, Autism, CBCL-DP, Emotion dysregulation, Twins, Aetiology

## Abstract

**Background:**

Emotion dysregulation (ED) is common in attention-deficit/hyperactivity disorder (ADHD) and often results in adverse outcomes. However, ED has been suggested as a transdiagnostic construct, why the specific association between ADHD and ED when adjusting for other mental health conditions needs further investigation. It is also important to determine the aetiological basis of the association between ADHD and ED to inform the theoretical conceptualization of ADHD.

**Method:**

This study used a co-twin control design, including a sample of dizygotic (DZ) and monozygotic (MZ) twins (N = 389; 45.8% females, age = 8–31 years, MZ twin pairs 57.6%). ED was assessed using the dysregulation profile from the parent-rated Child Behaviour Checklist and its adult version. Regression analyses were used across individuals and within the pairs, while adjusting for diagnoses of autism, intellectual disability, other neurodevelopmental conditions and affective conditions.

**Results:**

ADHD was significantly associated with ED, even when adjusting for age, sex, attention problems and other mental health conditions, and was the diagnosis most strongly associated with ED. Within-pair analyses revealed that twins with ADHD had higher levels of ED compared to their co-twin without ADHD. This association remained within DZ twins and was non-significant in the MZ subsample, with non-overlapping confidence intervals between the DZ and MZ estimates.

**Conclusion:**

ADHD is strongly and in part independently linked to ED, stressing the importance of early detection and treatment of emotional difficulties within this group. The findings from the within-pair analyses indicate a genetic influence on the association between ADHD and ED.

**Supplementary Information:**

The online version contains supplementary material available at 10.1186/s13034-022-00528-0.

## Background

Attention-deficit/hyperactivity disorder (ADHD) is one of the most common neurodevelopmental conditions (NDCs), affecting  ~ 5% of children, adolescents and adults worldwide [1]. ADHD is defined by symptoms of inattention and/or hyperactivity/impulsivity [2], which often persist into adulthood and are associated with work-related, academic and social challenges, as well as increased risk for other psychiatric problems [3]. Around 25–45% of children and 30–70% of adults with ADHD also have difficulties regulating emotions, resulting in more severe outcomes [4–7]. These emotional difficulties have been labelled in a variety of ways [8]. In the current study, we adopt a broad definition of emotion dysregulation (ED) including maladaptive emotional reactivity (i.e., experiential, behavioural and physiological responses) and emotion regulation (i.e., the process of altering these responses) [8, 9].

ED has in recent years been conceptualised as a pivotal transdiagnostic construct underlying several psychiatric diagnoses [10–12] and has frequently been operationalised by the dysregulation profile (DP) derived from the Child Behaviour Checklist (CBCL) [13]. The DP has been proposed to measure a general dysregulation factor [14–16]. Accordingly, the DP has been found to identify ED in several clinical populations, including ADHD, autism, anxiety and depression, as well as in non-clinical populations [16–20]. Despite recent research emphasising the transdiagnostic nature of ED, there is an ongoing debate regarding the role of ED in ADHD. Some researchers suggest that ED should be considered a core feature of ADHD since ED is common in individuals with ADHD, correlates with core ADHD symptoms and contributes to poor clinical outcomes [4, 21]. In addition, pharmacological treatments targeting ADHD have been found to alleviate difficulties with ED, although effects vary across studies [8].

Previous research investigating the relation between ADHD and ED are limited by a focus on male participants and by often excluding other mental health conditions, why firm conclusions about universality and specificity are hampered [22, 23]. Furthermore, the existing literature is often based on diagnostic classifications according to the Diagnostic and Statistical Manual of Mental Disorders 4th Edition (DSM-IV) [23, 24], which excludes the possibility of being diagnosed with both ADHD and autism. Because ADHD frequently co-occur with autism [25], previous research may have underestimated the role of co-occurring autism in ADHD when explaining ED. Corroborating this, a study examining the DP in youths with autism or ADHD found that individuals with autism were at higher risk of having severe ED than the ADHD group, although both conditions were associated with elevated ED [18]. Furthermore, other common co-occurring NDCs, such as specific learning disorders, motor problems and intellectual disability (ID), are rarely accounted for, even though ED is prominent in these conditions [10]. Such findings highlight the importance of taking other mental health conditions into account when investigating the association between ED and ADHD.

Given the ongoing debate regarding the relation between ED and ADHD, it is of particular interest to assess the aetiological basis for this association. Genetic factors are likely to play a crucial role, since ADHD is highly heritable [26] and emotion regulation, including ED as measured with the DP, has been shown to be influenced by genetic factors, too [27, 28]. Accordingly, previous studies have found that symptoms common in ED, such as irritability, share genetic risk factors with ADHD [29, 30]. Relatedly, a community-based twin study found that a majority of the aetiological overlap between symptoms of ADHD and ED in children and adolescents could be attributed to genetics [31]. However, this study did not use diagnostic interviews to assess diagnostic status and used a narrow measure of ED. Sobanski and colleagues [32] found no evidence for the influence of familial factors on the association between clinical ADHD and ED symptoms, since siblings to probands with ED had no increased risk for ADHD. Previous findings need to be expanded with a broader measure of ED, as well as a more in-depth investigation of genetic and environmental influences to disentangle the potentially shared aetiology of ADHD and ED [32].

In summary, while there is an established association between ED and ADHD, it remains unclear whether ED is independently related to ADHD when controlling for other mental health conditions, and the genetic and environmental influences desire clarification. It is also important to acknowledge developmental periods when studying ED since regulatory abilities have been shown to improve from age 10 up to late adolescence before tapering off [33]. Hence, utilizing a broader age span when investigating the association between ADHD and ED is important. To address the limitations in previous research, the current study used a co-twin control-study design in a deeply phenotyped sample of child, adolescent and adult dizygotic (DZ) and monozygotic (MZ) twin pairs, enriched for ADHD and other mental health conditions. We hypothesised that ADHD would remain associated with ED, although attenuated, when adjusting for other mental health conditions and that the relation between ADHD and ED would be influenced by genetic factors.

## Methods

### Participants and procedure

We used data from an ongoing project, the Roots of Autism and ADHD Twin Study in Sweden (RATSS; [34, 35]. RATSS includes twin pairs, mainly recruited from the population-based Child and Adolescent Twin Study in Sweden (CATSS) [36] and the Young Adult Twins in Sweden Study (YATSS) [37], in which one or both twins have screened positively for NDCs (e.g., via telephone interviews and surveys) and where typically developing (TD) twins in the same age range are recruited as control participants. During a 2½-day visit to a clinical research unit, participants were behaviourally and biologically phenotyped, as well as diagnostically assessed, as part of the RATSS study (for details, see [34]). In the current study, only same-sex twin pairs were included, which led to the exclusion of nine pairs and one group of triplets with opposite sex. In addition, four twin pairs were excluded due to missing data on the ED measure. One twin pair was excluded as one of the twins had a rare genetic syndrome. One family had two twin pairs included in RATSS and we excluded one of these pairs. In total, 33 twins from the RATSS cohort were a priori excluded.

The final sample consisted of 389 twins (224 MZ and 165 DZ, including a trio of DZ triplets). Zygosity was determined by single nucleotide polymorphism markers (for details, see [38]), or on physical appearance measured by a 4-item questionnaire (7.7% of the participants). Included diagnoses were ADHD, autism, other NDCs (specific learning, tic and speech/language disorders), affective conditions (mood and anxiety conditions, obsessive compulsive disorder and post-traumatic stress disorder) and ID. A majority of the individuals with ADHD were prescribed medication for the condition (*n* = 61, 57%). Note that a substantial proportion of participants had more than one diagnosis (*n* = 100), thus belonging to more than one of the diagnostic group categories in the statistical analyses. Being discordant for a mental condition meant that one of the twins in a pair had a diagnosis, while their co-twin did not. This study was approved by the National Swedish Ethical Review Board and written and oral informed consent was obtained from all participants. See Table 1 for sample characteristics.

**Table 1:** Study sample characteristics

	Total twin sample *(N* = *389)*	DZ twins *(n* = *165)*	MZ twins *(n* = *224)*
Sex (females), *N (%)*	178 (45.8%)	70 (42.4%)	108 (48.2%)
Years of age, *mean/range*	16.75/8–36	15.15/8–31	17.93/8–33
ADHD diagnosis, *N* (%)	107 (27.5%)	63 (38.2%)	44 (19.6%)
Autism diagnosis, *N* (%)	89 (22.9%)	42 (25.5%)	47 (21.0%)
Other NDC, *N* (%)	63 (16.2%)	36 (21.5%)	27 (12.1%)
Affective condition, *N* (%)	75 (19.3%)	35 (21.2%)	40 (17.9%)
ID, *N* (%)	19 (4.9%)	6 (3.6%)	13 (5.8%)
Typically developed, *N* (%)	172 (44.2%)	58 (35.2%)	114 (50.9%)
Qualitatively discordant for ADHD, *N (twin pairs)*	54	40	14
Qualitatively discordant for autism, *N (twin pairs)*	53	32	21
Qualitatively discordant for other NDCs, *N (twin pairs)*	36	25	11
Qualitatively discordant for affective conditions, *N (twin pairs)*	47	25	22
Qualitatively discordant for ID, *N (twin pairs)*	13	6	7

*ADHD* attention-deficit/hyperactivity disorder, *DZ* dizygotic, *MZ* monozygotic, *NDCs* neurodevelopmental conditions, *ID* intellectual disability

### Diagnostic assessments

Psychiatric diagnoses were based on the DSM-5 [2] and determined by a group of experienced clinicians during the participants visit to the clinical research unit. Diagnostic instruments included the Kiddie Schedule for Affective conditions and Schizophrenia; the Structured Clinical Interview for DSM-IV (axis 1); ADHD-specific instruments such as the Diagnostic Interview for ADHD in adults; autism-specific instruments such as the Autism Diagnostic Observation Schedule Second Edition, the Autism Diagnostic Interview-Revised and the Social Responsiveness Scale Second Edition; adaptive functioning using the Adaptive Behavior Assessment System-2. General intellectual ability was based on results from the Wechsler Intelligence Scales for Children or Adults, or a combination of Leiter scales and the Peabody Picture Vocabulary test depending on the participant’s verbal abilities. For more detail regarding diagnostic assessments, see [34].

### Emotion dysregulation

ED was measured by the DP based on parent-ratings from the Achenbach System of Empirically Based Assessment (ASEBA) [13]. The DP targets different aspects of physiological, behavioural and cognitive regulatory difficulties in regards to emotions. It consists of three syndrome scales of the CBCL, encompassing externalising and internalising symptoms: the anxious/depressed, aggressive behaviour and attention problems subscales [12, 20]. On each subscale, items are rated on a scale from 0 to 2 (0 = not true, 1 = somewhat or sometimes true and 2 = very true or often true) [13]). In accordance with the ASEBA manual, raw total scores on each syndrome scale were converted to normative t-scores (M = 50, SD = 10 ±), where a t-score of  ≤ 50 is defined as the starting point for each subscale and t-scores of ≥ 65 are deemed clinical or probably clinical levels [13]. The DP is calculated by summing the t-scores of each subscale [20]. The summed t-score was defined as the measure for ED, where higher scores indicate increasing dysregulation. A summed t-score of  < 180 indicate no ED, a score between 180 and  < 210 indicate moderate ED and a t-score  ≥ 210 indicate severe ED [17]. A matching profile for the Adult Behaviour Checklist (ABCL) [39] was created for adult twins (≥ 18 years old), consistent with a previous study on ED in adults [40].

### Statistical analyses

Conditional linear regression analyses within the generalized estimating equations framework using the “drgee” package (v.1.1.10) in R (v. 4.0.5) were used [41, 42]. All exposure variables in the main analyses, besides age, were dichotomised categorical variables (diagnosis or sex), whereas the outcome variable (ED) was defined as a continuous variable. The analyses were conducted in several steps. First, we estimated associations between ADHD diagnosis and ED across the entire sample, while adjusting for age and sex, where twins were treated as separate individuals but standard errors were adjusted for twin clustering. In step two, we adjusted for other specific diagnostic groups (other NDCs, ID and affective conditions), also defined as dichotomous categorical variables. Second, we repeated the aforementioned steps for the within-pair analyses to adjust for unmeasured familial confounders. In these within-pair analyses, the difference in the exposure variable within a pair is correlated with the difference in the outcome variable within the same pair. No adjustments for sex or age were required because each twin pair was examined at the same time and twin pairs with differing sex were a priori excluded. Third, we re-calculated the within-pair analyses in the DZ and MZ sub-cohorts in order to investigate aetiological influences. By design, we implicitly adjusted for shared environmental factors, as well as half of the genetic factors in the DZ sample and 100% of genetics in the MZ sample. Therefore, any remaining associations between the exposure (diagnosis) and the outcome (ED) in the MZ sample could only be attributed to factors differentiating the twins, which is non-shared environmental influences [43]. Two tailed tests with *p* values  < 0.05 were considered significant.

Because the DP includes a subscale of self-regulatory challenges in attention capacity, and symptoms of inattention are part of the diagnostic criteria for ADHD, any associations found between ADHD and ED could be attributed to this overlap of inattention symptoms. Due to this potential bias, as a post-hoc analysis, we re-calculated the analyses while excluding participants who had an ADHD diagnosis with elevated t-scores only on the attention subscale and not on the other subscales of the DP (*n* = *11*). We also re-calculated the analyses while excluding the attention subscale from our outcome measure, thus only including the summed t-scores of the aggression and anxious/depressed subscales.

## Results

Among participants with NDCs, a majority scored in the moderate to severe ED range: 62.7% with ADHD, 56.1% with autism, 63.3% in ID and 57.2% in participants with other NDCs. Among participants with affective conditions, 38.7% showed scores in the moderate to severe ED range, whereas 5.8% of those without any NDC or affective condition had ED. See Table 2 for descriptive statistics.

**Table 2:** Descriptive statistics over the distribution of ED across the entire sample and different sub-groups

	ED *Mean t-score (SD)*	No ED *(t-score:* < *180) N (%)*	Moderate ED *(t-score:* ≥ *180–* < *210) N (%)*	Severe ED *(t-score:* ≥ *210) N (%)*
Entire sample	168.40 (21.09)	286 (73.5%)	82 (21.1%)	21 (5.4%)
Sex				
Females	168.48 (23.05)	130 (73.0%)	35 (19.7%)	13 (7.3%)
Males	168.34 (19.35)	156 (73.9%)	47 (22.3%)	8 (3.8%)
ADHD	189.05 (23.00)	40 (37.4%)	48 (44.9%)	19 (17.8%)
Autism	183.49 (21.94)	39 (43.8%)	40 (44.9%)	10 (11.2%)
Other NDCs	183.08 (23.50)	27 (42.9%)	27 (42.9%)	9 (14.3%)
Affective conditions	175.52 (24.18)	46 (61.3%)	20 (26.7%)	9 (12.0%)
ID	184.05 (19.54)	7 (36.8%)	11 (57.9%)	1 (5.3%)
TD	156.56 (10.97)	162 (94.2%)	9 (5.2%)	1 (0.6%)

Emotion dysregulation (*ED*) as measured by the sum of t-scores on the dysregulation profile; *ADHD* attention-deficit/hyperactivity disorder, *NDCs* neurodevelopmental conditions, *ID* intellectual disability, *TD* typically developing

### Across individuals

As shown in Table 3, having an ADHD diagnosis was associated with higher levels of ED in the non-adjusted model and when adjusting for age and sex. In addition, female sex and a lower age were associated with higher levels of ED. Despite adjusting for autism diagnosis, ID, other NDCs and affective conditions, the association between ADHD and ED remained. In the final model, all diagnoses, except for ID, were associated with higher levels of ED.

**Table 3:** Associations with ED across individuals for the entire sample

	Non-adjusted model *ED (N* = *389)*				Adjusted model 1 *ED (N* = *389)*				Adjusted model 2 *ED (N* = *389)*			
	*b*	95% CI	*SE*	*Z*	*b*	95% CI	*SE*	Z	*b*	95% CI	*SE*	*Z*
ADHD	28.48***	23.03–33.93	2.78	10.24	25.79***	20.68–30.90	2.61	9.90	20.75***	15.73–25.76	2.56	8.10
Age					− 0.84***	− 1.13 to − 0.56	0.15	− 5.81	-0.88***	− 1.15 to − 0.61	0.14	− 6.42
Sex (female)					5.48**	1.38–9.57	2.09	2.62	5.31**	1.50–9.12	1.94	2.73
Autism									9.57***	4.87–14.28	2.40	3.99
Other NDCs									6.93**	1.78–12.09	2.63	2.64
ID									6.16	− 1.57–13.89	3.95	1.56
Affective conditions									8.94***	4.37–13.52	2.33	3.83

Bold indicate p < 0.05; *** = *p* < 0.001, ** = *p* < 0.01; Emotion dysregulation (*ED*) as measured by the sum of t-scores in the dysregulation profile

*ADHD*, attention-deficit/hyperactivity disorder; *NDCs*, neurodevelopmental conditions, *ID*, intellectual disability

### Within-pairs associations

As shown in Table 4, the twins with ADHD diagnoses had higher levels of ED compared to their co-twins without ADHD, even when adjusting for other diagnoses. Having an autism diagnosis or an affective condition was associated with higher levels of ED as compared to the co-twin without a diagnosis in the adjusted model. In contrast to the analyses across individuals, the relation between other NDCs and ED was not significant. Within DZ twin pairs, the only association that remained was between ADHD and ED. Within the MZ sample, the relation between ADHD and ED was attenuated in the non-adjusted model and non-significant in the adjusted model, whereas the association between autism and ED, and affective conditions and ED, were significant.

**Table 4:** Within-pairs associations with ED across the entire sample and in DZ and MZ sub-cohorts

	Total sample *ED (N* = *194 twin pairs)*				DZ sample *ED (n* = *82 twin pairs)*				MZ sample *ED (n* = *112 twin pairs)*			
	*b*	95% CI	*SE*	*Z*	*b*	95% CI	*SE*	*Z*	*b*	95% CI	*SE*	*Z*
Unadjusted model												
ADHD	22.70***	16.31–29.09	3.26	6.96	26.31***	18.66–33.97	3.91	6.74	12.29*	2.73–21.84	4.88	2.52
Adjusted model												
ADHD	20.41***	13.84–26.98	3.35	6.09	25.29***	17.74–32.85	3.86	6.56	4.61	− 2.25–11.47	3.50	1.32
Autism	6.55*	0.06–13.05	3.31	1.98	2.18	− 7.76–12.12	5.07	0.43	14.72***	7.48–21.95	3.70	3.99
Other NDCs	3.91	− 2.65–10.46	3.34	1.17	4.76	− 4.58–14.10	4.77	1.00	4.14	− 0.32–8.60	2.27	1.82
ID	3.18	− 7.42–13.77	5.41	0.59	2.96	− 13.64–19.55	8.47	0.35	7.50	− 3.53–18.52	5.63	1.33
Affective conditions	6.28*	0.77–11.79	2.81	2.23	3.98	− 4.80–12.76	4.48	0.89	8.10**	2.89–13.31	2.66	3.05

Bold indicate p < 0.05; *** = *p* < 0.001, ** = *p* < 0.01, * = *p* < 0.05; Emotion dysregulation (*ED*) as measured by the sum of t-scores in the dysregulation profile

*ADHD* attention-deficit/hyperactivity disorder, *DZ* dizygotic, *MZ* monozygotic, *NDCs* neurodevelopmental conditions, *ID* intellectual disabilit

### Post-hoc analyses

When excluding participants with an ADHD diagnosis and elevated t-scores only on the attention subscale, the same pattern emerged as in the initial analyses, see Additional file 1: Table S1 and Table S2. When excluding the attention subscale from the outcome measure, the results did not differ significantly, see Additional file 1: Table S3 and Table S4.

## Discussion

We investigated the association between ADHD and ED across individuals and within DZ and MZ twin pairs. ADHD was associated with higher levels of ED across the entire sample, even when adjusting for other mental health conditions. In fact, ADHD was the diagnosis most strongly associated with ED. These findings indicate that ADHD is in part independently related to ED. Post-hoc analyses further suggest that the relationship cannot be explained solely by the overlap of inattention symptoms present in both ADHD and ED. As the relation between ADHD and ED was significant in the DZ twin pairs but non-significant in the MZ sub-cohort, with non-overlapping confidence intervals between the DZ and MZ estimates in the final model, these findings suggest a genetic influence on the association.

A majority of the twins with NDCs, including ADHD and autism, had a dysregulation profile indicating moderate to severe ED. This supports the notion of ED being a construct of transdiagnostic value across several mental health conditions [10, 12] and endorses the meaningfulness of investigating dimensional latent constructs in mental health in addition to categorically defined psychiatric entities, e.g., as suggested by the NIMH Research Domains Frame-work (RDoC) [44, 45]. In line with our hypothesis, ADHD remained associated with ED when we adjusted for other mental health conditions. Even though ED was associated with other diagnoses, ADHD was the diagnosis most strongly linked to ED and overrepresented in the severe ED group, suggesting that individuals with ADHD may be a particularly vulnerable group at risk for ED. The association was evident despite that the majority of the participants with ADHD were prescribed medication for the condition, which usually positively affects ED [8]. This finding contradicts a previous study examining the DP in both autism and ADHD, where autism was more strongly linked with severe ED than ADHD [18]. However, a major difference between this study and ours is that we implicitly adjusted for genetic and shared environmental factors in the twins, which might explain the different results.

We further found that female sex was associated with higher levels of ED. Previous reports on potential sex differences with regard to ED are mixed, both in the general population and in ADHD [5, 32, 46, 47]. Methodological differences, such as various definitions of ED, differing age-spans and study populations, may account for these inconsistent findings, which warrants further research. We also found that ED was more common in younger participants in the analyses across individuals. This is consistent with other studies indicating that youth is a turbulent period regarding emotional experiences [46] and that emotion-regulation capacity may mature, despite stable levels of emotional reactivity [33]. However, it is also possible that the effect of age on ED is not linear, and research focusing on specific developmental periods or age differences regarding ED is scarce, needing further scrutiny [48].

As hypothesised, the relation between ADHD and ED was influenced by genetic factors. This finding supports previous research indicating that shared genetic factors may explain the link between ADHD and ED [29–31] and expands previous literature by applying a co-twin control design across different developmental periods while also controlling for other mental health conditions. Regarding the ongoing debate surrounding the role of ED in ADHD, the strong association found between ADHD and ED endorse the notion of ED being a core feature of ADHD. At the same time, when taking both genetic and environmental familial factors into consideration, the association between the two is lost, hence indicating that it is not ADHD per se that predicts ED, but rather a common genetic background that influences both variables. However, since we used a broad measure of ED, we were unable to investigate the association between ADHD and specific aspects of ED. Relatedly, it has been hypothesised that distinct presentations of ED might differ between ADHD subtypes [8] why more research into this topic is needed. Another finding was that the relation between autism and ED, as well as between affective conditions and ED, was significant in the MZ sample, indicating a non-shared influence on the associations. These effects may be masked by confounding factors between twin pairs, such as genetics and shared environment, which might explain why the associations were non-significant in DZ twins.

## Limitations and strengths

When splitting the sample into MZ and DZ twin pairs, the risk of being statistically underpowered increases. Moreover, the smaller amount of MZ twin pairs being discordant for ADHD, as compared to the DZ sub-cohort, may result in lower power and influence the within-pairs analyses. At the same time, for twin pairs discordant for ADHD in the within-pairs analyses, the confidence intervals are non-overlapping between the DZ and MZ sub-cohorts in the adjusted model, justifying our conclusion of genetic confounding. A larger sample would enable further examination of the associations within other relevant sub-cohorts, such as different age groups and medication-naïve individuals. ED was assessed using parental ratings, which may be subject to bias, and although the DP is a well-established measure of general dysregulation [14–16], it was not originally developed to specifically measure ED [49]. Therefore, it would have been interesting to include additional and more objective measures targeting ED to increase specificity. Moreover, our main exposure variable (ADHD) and part of the outcome measure (the attention subscale) are closely linked. Due to this potential bias, we added two post-hoc analyses which suggests that the relation between ADHD and ED cannot be attributed solely to inattention symptoms overlap. At the same time, emotion-regulation capacity may partly depend on attention-allocation processes [6]. Therefore, fully excluding this overlap may be misleading as these symptoms may reflect shared underlying mechanisms in ADHD and ED. However, inattention could play a more important role for the phenotypical presentation of ED in ADHD than in other mental health conditions, and, hypothetically, if we had used another measure of ED the association with other NDCs, such as autism, might have been stronger. In addition, the DP based on the ABCL has not been as rigorously studied as the CBCL-DP. The number of items of the different subscales differ between the CBCL and ABCL questionnaire (e.g., 10 versus 17 items in the attention subscale), which could have resulted in bias. On balance, the ABCL-DP version has been used in a previous study [40], and many of the items have the same wording in both questionnaires.

The strengths of the study include a rare and relatively large sample of DZ and MZ twin pairs both concordant and discordant for ADHD, other NDCs and affective conditions, allowing other mental health conditions to be taken into account. The sample include a considerable number of DZ and MZ twins in Sweden concordant and discordant for NDCs (for an overview, see [50]). The sample was thoroughly phenotyped and the participants underwent a comprehensive assessment by experienced clinicians strengthening the validity of the diagnostic evaluation. The co-twin control design is a powerful method that implicitly controls for familial confounders, age and sex [43, 51].

## Conclusions

The current study extends previous research into the relation between ADHD and ED by using a co-twin control-study design in a sample of deeply phenotyped children, adolescents and adults, enriched for ADHD and other mental health conditions. Our findings suggest that ED is in part independently related to ADHD and that individuals with ADHD seem to be a particularly vulnerable group for ED. Therefore, clinically it appears crucial to address difficulties with ED as a target when developing and evaluating both behavioural and pharmacological treatments in ADHD. In addition, genetic factors seem to influence the relation between ADHD and ED. Future studies should examine the genetic association in more depth to further increase understanding of potentially shared underlying mechanisms. Finally, in order to strengthen the likelihood of early detection of ADHD and develop more stratified treatments, there is a need to further specify how different aspects of ED is characterised in ADHD across development and how this may vary depending on sex, age and ADHD symptoms.

### Supplementary Information

**Additional file 1:** **Table S1.** Post-hoc analysis for associations across individuals where participants with ADHD and elevated t-scores on the attention subscale only were excluded. **Table S2.** Post-hoc analysis for within-pairs associations where participants with ADHD and elevated t-scores on the attention subscale only were excluded. **Table S3.** Post-hoc analysis for associations across individuals where only items from the aggression and anxious/depressed subscales of the DP were included as the outcome measure. **Table S4.** Post-hoc analysis for within-pairs associations where only items from the aggression and anxious/depressed subscales of the DP were included as the outcome measure.

## Data Availability

The datasets presented in this article are not readily available because of the regulations in the ethical approval and university policies, requiring among others a data sharing agreement. Requests to access the datasets should be directed to sven.bolte@ki.se.

## Abbreviations

ABCL: Adult Behaviour Checklist
ADHD: Attention-deficit/hyperactivity disorder
CATSS: Child and Adolescent Twin Study in Sweden
CBCL: Child Behaviour Checklist
DP: Dysregulation profile
DSM: Diagnostic and Statistical Manual of Mental Disorders
DZ: Dizygotic
ED: Emotion dysregulation
ID: Intellectual disability
MZ: Monozygotic
NDC: Neurodevelopmental conditions
RATSS: Roots of Autism and ADHD Twin Study in Sweden
RDoC: Research Domains Frame-work
TD: Typically developing
