# Correction: Emotion dysregulation in ADHD and other neurodevelopmental conditions: a co-twin control study

**DOI:** 10.1186/s13034-024-00709-z

**Published:** 2024-02-12

**Authors:** Rebecka Astenvald, Matilda A. Frick, Janina Neufeld, Sven Bölte, Johan Isaksson

**Affiliations:** 1 Department of Medical Sciences, Child and Adolescent Psychiatry Unit, Uppsala University, Uppsala, Sweden; 2 Department of Psychology, Division of Emotion Psychology, Uppsala University, Uppsala, Sweden; 3 Center of Neurodevelopmental Disorders (KIND), Centre for Psychiatry Research, Department of Women’s and Children’s Health, Karolinska Institutet & Stockholm Health Care Services, Stockholm, Sweden; 4 Swedish Collegium for Advanced Study (SCAS), Uppsala, Sweden; 5 Curtin Autism Research Group, Curtin School of Allied Health, Curtin University, Perth, WA Australia; 6 Child and Adolescent Psychiatry, Stockholm Health Care Services, Stockholm, Sweden

**Correction: Child and Adolescent Psychiatry and Mental Health (2022) 16:92** 10.1186/s13034-022-00528-0

Following publication of the original article [1], an error in data handling was discovered. The error had no significant effect on the results, interpretations, discussions or any of the conclusions drawn from the study.

A total of seven participants (1.8% of the total sample) were included in the final sample by mistake. These participants had complete missing data on the outcome measure, which was erroneously recorded as zeros when computing the variables for the subscales. This mishap affected approximately 0.7% of the ABCL data and 2.4% of the CBCL data. The re-calculations altered the data within the tables and reduced the number of included participants from N = 389 to N = 382 (45.5% females, age = 8–36 years, MZ twin pairs 58.4%).

For the main analyses and findings, updated estimates are presented below:

Across individuals, the associations between ADHD and higher levels of emotion dysregulation in the non-adjusted model changed from *b* = 28.48 (*SE* = 2.78) to *b* = 29.45 (*SE* = 2.77), from *b* = 25.79 (*SE* = 2.61) to *b* = 26.89 (*SE* = 2.57) when adjusting for age and sex, and from *b* = 20.75 (*SE* = 2.56) to *b* = 21.48 (*SE* = 2.51) when adjusting for other psychiatric conditions. The difference in beta-values between the initial analyses and the re-calculations ranged from 0.73–1.10. Overall, the beta-values showed a slightly stronger association between ADHD and emotion dysregulation in the re-calculations.

For the within-pair analyses, in the fully adjusted model, the associations between ADHD and higher levels of emotion dysregulation changed from *b* = 20.41 (*SE* = 3.35, n = 194 twin pairs) to *b* = 22.43 (*SE* = 3.26, n = 188 twin pairs), and from *b* = 25.29 (*SE* = 3.86, n = 82 twin pairs) to *b* = 28.29 (*SE* = 3.67, n = 77 twin pairs) in the DZ sub cohort. The association between ADHD and emotion dysregulation remained non-significant in the MZ sub cohort and estimates changed from *b* = 4.61 (*SE* = 3.50, n = 112 twin pairs) to *b* = 4.59 (*SE* = 3.50, n = 111 twin pairs) in the re-calculations. The difference in beta-values between the initial analyses and the re-calculations ranged from 0.02–3.00.

All data from the re-calculations are available upon request from the corresponding author.
