# Supplementary material for: Emotion dysregulation in ADHD and other neurodevelopmental conditions: a co-twin control study
Source: Child Adolesc Psychiatry Ment Health. 2022 Nov 28;16:92. doi: 10.1186/s13034-022-00528-0 (PMC9706824; doi:10.1186/s13034-022-00528-0)
Supplement: Supplementary file 1 — Additional file 1: Table S1. Post-hoc analysis for associations across individuals where participants with ADHD and elevated t-scores on the attention subscale only were excluded. Table S2. Post-hoc analysis for within-pairs associations where participants with ADHD and elevated t-scores on the attention subscale only were excluded. Table S3. Post-hoc analysis for associations across individuals where only items from the aggression and anxious/depressed subscales of the DP were included as the outcome measure. Table S4. Post-hoc analysis for within-pairs associations where only items from the aggression and anxious/depressed subscales of the DP were included as the outcome measure. [file 13034_2022_528_MOESM1_ESM.docx]

Additional file information

| **Table S1** Post-hoc analysis for associations across individuals where participants with ADHD and elevated t-scores on the attention subscale only were excluded *(n=11).* | | | | | | | | | | | | | | | |
| --- | --- | --- | --- | --- | --- | --- | --- | --- | --- | --- | --- | --- | --- | --- | --- |
|  | Non-adjusted model  *ED*  *(N = 378)* | | | |  | Adjusted model 1  *ED*  *(N = 378)* | | | | | Adjusted model 2  *ED*  *(N = 378)* | | | | |
|  | b | 95% CI | *SE* | *Z* |  | b | 95% CI | *SE* | *Z* |  | b | 95% CI | *SE* | *Z* |  |
| ADHD | **29.02***** | 23.11 – 34.92 | 3.01 | 9.63 |  | **26.34***** | 20.91 – 31.77 | 2.77 | 9.51 |  | **21.39***** | 16.09 – 26.69 | 2.70 | 7.91 |  |
| Age |  |  |  |  |  | **-0.87***** | -1.16 – -0.59 | 0.15 | -6.02 |  | **-0.90***** | -1.17 – -0.62 | 0.14 | -6.46 |  |
| Sex (female) |  |  |  |  |  | **5.60**** | 1.46 – 9.73 | 2.11 | 2.66 |  | **5.44**** | 1.57 – 9.31 | 1.98 | 2.75 |  |
| Autism |  |  |  |  |  |  |  |  |  |  | **9.99***** | 5.13 – 14.85 | 2.48 | 4.03 |  |
| Other NDCs |  |  |  |  |  |  |  |  |  |  | **6.65*** | 1.37 – 11.94 | 2.70 | 2.47 |  |
| ID |  |  |  |  |  |  |  |  |  |  | 7.27 | -0.54 – 15.07 | 3.98 | 1.82 |  |
| Affective conditions |  |  |  |  |  |  |  |  |  |  | **8.87***** | 4.26 – 13.48 | 2.35 | 3.77 |  |
| *Note:* Bold indicate p<0.05; *** = *p* <0.001, ** = *p* <0.01, * = *p* <0.05; Emotion dysregulation (ED) as measured by the sum of t-scores in the dysregulation profile; ADHD: attention-deficit/hyperactivity disorder; NDCs: neurodevelopmental conditions, ID: intellectual disability | | | | | | | | | | | | | | | |

| **Table S2** Post-hoc analysis for within-pairs associations where participants with ADHD and elevated t-scores on the attention subscale only were excluded *(n=11).* | | | | | | | | | | | | | | | |
| --- | --- | --- | --- | --- | --- | --- | --- | --- | --- | --- | --- | --- | --- | --- | --- |
|  | Entire sample *ED*  *(N = 183 twin pairs)* | | | |  | DZ sample  *ED*  *(n = 76 twin pairs)* | | | | | MZ sample  *ED*  *(n = 107 twin pairs)* | | | | |
|  | b | 95% CI | *SE* | *Z* |  | b | 95% CI | *SE* | *Z* |  | b | 95% CI | *SE* | *Z* |  |
| Unadjusted model |  |  |  |  |  |  |  |  |  |  |  |  |  |  |  |
| ADHD | **22.66***** | 15.84 – 29.48 | 3.48 | 6.51 |  | **26.02***** | 17.78 – 34.26 | 4.20 | 6.19 |  | **13.00*** | 2.81 – 23.19 | 5.20 | 2.50 |  |
| Adjusted model |  |  |  |  |  |  |  |  |  |  |  |  |  |  |  |
| ADHD | **20.17***** | 13.19 – 27.16 | 3.56 | 5.67 |  | **24.77***** | 16.69 – 32.85 | 4.12 | 6.01 |  | 4.41 | -3.14 – 11.96 | 3.85 | 1.15 |  |
| Autism | 6.92 | -0.17 – 14.00 | 3.62 | 1.91 |  | 2.69 | -8.34 – 13.73 | 5.63 | 0.48 |  | **15.21***** | 7.61 – 22.81 | 3.88 | 3.92 |  |
| Other NDCs | 4.33 | -2.42 – 11.07 | 3.44 | 1.26 |  | 5.20 | -4.92 – 15.33 | 5.17 | 1.00 |  | 3.99 | -0.42 – 8.40 | 2.25 | 1.77 |  |
| ID | 3.29 | -8.65 – 15.24 | 6.10 | 0.54 |  | 2.78 | -14.78 – 20.33 | 8.96 | 0.31 |  | 8.22 | -4.97 – 21.40 | 6.73 | 1.22 |  |
| Affective conditions | 5.10 | -0.91 – 11.11 | 3.07 | 1.66 |  | 3.37 | -6.07 – 12.80 | 4.81 | 0.70 |  | **6.46*** | 0.90 – 12.03 | 2.84 | 2.27 |  |
| *Note:* Bold indicate p<0.05; *** = *p* <0.001, ** = *p* <0.01, * = *p* <0.05; Emotion dysregulation (ED) as measured by the sum of t-scores in the dysregulation profile; ADHD: attention-deficit/hyperactivity disorder; DZ: dizygotic; MZ: monozygotic; NDCs: neurodevelopmental conditions, ID: intellectual disability | | | | | | | | | | | | | | | |

| **Table S3** Post-hoc analysis for associations across individuals where only items from the aggression and anxious/depressed subscales of the DP were included as the outcome measure. | | | | | | | | | | | | | | | |
| --- | --- | --- | --- | --- | --- | --- | --- | --- | --- | --- | --- | --- | --- | --- | --- |
|  | Non-adjusted model  *ED*  *(N = 389)* | | | |  | Adjusted model 1  *ED*  *(N = 389)* | | | | | Adjusted model 2  *ED*  *(N = 389)* | | | | |
|  | b | 95% CI | *SE* | *Z* |  | b | 95% CI | *SE* | *Z* |  | b | 95% CI | *SE* | *Z* |  |
| ADHD | **16.02***** | 12.25 – 19.79 | 1.92 | 8.33 |  | **14.43***** | 10.91 – 17.95 | 1.80 | 8.04 |  | **11.25***** | 7.63 – 14.87 | 1.85 | 6.09 |  |
| Age |  |  |  |  |  | **-0.53***** | -0.74 – -0.33 | 0.10 | -5.21 |  | **-0.57***** | -0.77 – -0.37 | 0.10 | -5.63 |  |
| Sex (female) |  |  |  |  |  | **4.37**** | 1.41 – 7.34 | 1.51 | 2.89 |  | **4.14**** | 1.31 – 6.98 | 1.45 | 2.86 |  |
| Autism |  |  |  |  |  |  |  |  |  |  | **5.49**** | 2.14 – 8.84 | 1.71 | 3.21 |  |
| Other NDCs |  |  |  |  |  |  |  |  |  |  | **5.15*** | 1.14 – 9.16 | 2.05 | 2.52 |  |
| ID |  |  |  |  |  |  |  |  |  |  | 1.24 | -3.71 – 6.18 | 2.52 | 0.49 |  |
| Affective conditions |  |  |  |  |  |  |  |  |  |  | **6.75***** | 3.44 – 10.07 | 1.69 | 3.99 |  |
| *Note:* Bold indicate p<0.05; *** = *p* <0.001, ** = *p* <0.01, * = *p* <0.05; Emotion dysregulation (ED) as measured by the sum of t-scores in the dysregulation profile (DP); ADHD, attention-deficit/hyperactivity disorder; NDCs, neurodevelopmental conditions, ID, intellectual disability | | | | | | | | | | | | | | | |

| **Table S4** Post-hoc analysis for within-pairs associations where only items from the aggression and anxious/depressed subscales of the DP were included as the outcome measure. | | | | | | | | | | | | | | | |
| --- | --- | --- | --- | --- | --- | --- | --- | --- | --- | --- | --- | --- | --- | --- | --- |
|  | Entire sample  *ED*  *(N = 194 twin pairs)* | | | |  | DZ sample  *ED*  *(n = 82 twin pairs)* | | | | | MZ sample  *ED*  *(n = 111 twin pairs)* | | | | |
|  | b | 95% CI | *SE* | *Z* |  | b | 95% CI | *SE* | *Z* |  | b | 95% CI | *SE* | *Z* |  |
| Unadjusted model |  |  |  |  |  |  |  |  |  |  |  |  |  |  |  |
| ADHD | **11.65***** | 6.76 – 16.54 | 2.49 | 4.67 |  | **14.01***** | 8.43 – 19.59 | 2.85 | 4.92 |  | 4.86 | -4.40 – 14.12 | 4.72 | 1.03 |  |
| Adjusted model |  |  |  |  |  |  |  |  |  |  |  |  |  |  |  |
| ADHD | **9.94***** | 5.24 – 14.64 | 2.40 | 4.15 |  | **13.08***** | 8.44 – 18.71 | 2.63 | 4.98 |  | -0.11 | -6.91 – 6.70 | 3.47 | -0.03 |  |
| Autism | **5.25*** | 0.66 – 9.82 | 2.34 | 2.24 |  | 1.37 | -6.18 – 8.16 | 3.63 | 0.38 |  | **11.56***** | 6.05 – 17.06 | 2.81 | 4.12 |  |
| Other NDCs | 3.60 | -1.89 – 9.09 | 2.80 | 1.29 |  | 5.65 | -1.61 – 14.71 | 4.09 | 1.38 |  | 0.79 | -2.45 – 4.03 | 1.65 | 0.48 |  |
| ID | -0.55 | -7.87 – 6.78 | 3.74 | -0.15 |  | 2.35 | -7.75 – 13.09 | 5.33 | 0.44 |  | -0.30 | -9.38 – 8.77 | 4.63 | -0.07 |  |
| Affective conditions | **5.33**** | 1.34 – 9.31 | 2.03 | 2.63 |  | 3.75 | -2.79 – 9.86 | 3.20 | 1.17 |  | **5.99**** | 1.78 – 10.20 | 2.15 | 2.79 |  |
| *Note:* Bold indicate p<0.05; *** = *p* <0.001, ** = *p* <0.01, * = *p* <0.05; Emotion dysregulation (ED) as measured by the sum of t-scores in the dysregulation profile (DP); ADHD, attention-deficit/hyperactivity disorder; DZ, dizygotic; MZ, monozygotic; NDCs, neurodevelopmental conditions, ID, intellectual disability | | | | | | | | | | | | | | | |
